# Tobacco smoking and the risk of sudden cardiac death: a systematic review and meta-analysis of prospective studies

**DOI:** 10.1007/s10654-017-0351-y

**Published:** 2018-02-07

**Authors:** Dagfinn Aune, Sabrina Schlesinger, Teresa Norat, Elio Riboli

**Affiliations:** 1 Department of Epidemiology and Biostatistics, School of Public Health, Imperial College London, St. Mary’s Campus, Norfolk Place, Paddington, London, W2 1PG UK; 2 Bjørknes University College, Oslo, Norway; 3 Department of Endocrinology, Morbid Obesity and Preventive Medicine, Oslo University Hospital, Oslo, Norway; 4 Institute for Biometrics and Epidemiology, German Diabetes Center (DDZ) at Heinrich Heine University Düsseldorf, Düsseldorf, Germany

**Keywords:** Smoking, Sudden cardiac death, Systematic review, Meta-analysis

## Abstract

Smoking is an established risk factor for cardiovascular disease including coronary heart disease and stroke, however, data regarding smoking and sudden cardiac death have not been summarized in a meta-analysis previously. We therefore conducted a systematic review and meta-analysis to clarify this association. We searched the PubMed and Embase databases for studies of smoking and sudden cardiac death up to July 20th 2017. Prospective studies were included if they reported adjusted relative risk (RR) estimates and 95% confidence intervals (CIs) for smoking and sudden cardiac death. Summary RRs were estimated by use of a random effects model. Twelve prospective studies were included. The summary RR was 3.06 (95% CI 2.46–3.82, I^2^ = 41%, p_heterogeneity_ = 0.12, n = 7) for current smokers and 1.38 (95% CI 1.20–1.60, I^2^ = 0%, p_heterogeneity_ = 0.55, n = 7) for former smokers compared to never smokers. For four studies using non-current (never + former) smokers as the reference category the summary RR among current smokers was 2.08 (95% CI 1.70–2.53, I^2^ = 18%, p_heterogeneity_ = 0.30). The results persisted in most of the subgroup analyses. There was no evidence of publication bias. These results confirm that smoking increases the risk of sudden cardiac death. Any further studies should investigate in more detail the effects of duration of smoking, number of cigarettes per day, pack-years, and time since quitting smoking and sudden cardiac death.

**Electronic supplementary material:**

The online version of this article (10.1007/s10654-017-0351-y) contains supplementary material, which is available to authorized users.

## Introduction

Cardiovascular disease is the leading cause of death globally, accounting for 17.9 million deaths worldwide in 2015 [1]. It has been estimated that approximately 40–50% of all cardiovascular deaths are sudden cardiac deaths and about 80% of these are ventricular tachyarrhythmias [2]. In the US approximately 250 000–310 000 sudden cardiac deaths occur annually [3, 4]. Sudden cardiac death is defined as an unexpected, pulseless condition attributable to a cardiac arrhythmia [5], and most cardiac arrests present without warning symptoms and are usually fatal [6, 7]. Preventive efforts have focused on using cardioverter-defibrillators in the highest risk groups such as patients with advanced cardiomyopathy and reduced left ventricular ejection fraction [8], however, these high risk groups only account for 25–30% of all sudden cardiac deaths and the majority occur in the general population and in persons without established coronary heart disease [9, 10]. Population-wide strategies for primary prevention may therefore be a more promising approach to reduce the incidence of sudden cardiac deaths.

Established or suspected risk factors for sudden cardiac death include age, obesity, diabetes, physical inactivity, dietary factors, hypertension, high serum cholesterol, high resting heart rate and family history of sudden cardiac death [11–13]. A number of cohort studies have also reported a strong increase in the risk of sudden cardiac death among smokers [13–23], however, the strength of the associations reported have varied from a 50% increase in risk to 5.5-fold increases in risk [13–23]. Differences in effect sizes may be due to differences in; the sample sizes, duration of follow-up, geographic location, or the definition of the reference group (e.g. never smokers vs. non-current smokers) between studies and/or chance variation. To our knowledge there has not been published a meta-analysis on smoking and sudden cardiac death previously. For these reasons we conducted a systematic review and meta-analysis of published prospective studies on smoking and risk of sudden cardiac death. Specifically we aimed to clarify the strength of the association, the shape of the dose–response relationship, the effects of quitting smoking and potential sources of heterogeneity in subgroup and sensitivity analyses.

## Materials and methods

### Search strategy and inclusion criteria

We searched the Pubmed, and Embase databases from their inception up to July 20th 2017 for eligible studies (DA, SS). The search terms used are shown in Supplementary Table 1 and 2. We followed standard criteria (Moose criteria) for reporting meta-analyses [24]. In addition, we searched the reference lists of the identified publications for further studies. Study quality was assessed using the Newcastle–Ottawa scale which rates studies according to selection, comparability and outcome assessment with a score range from 0 to 9 [25].

### Study selection

Eligible studies for inclusion in the meta-analysis included published retrospective and prospective cohort studies and nested case–control studies within cohorts that investigated the association between smoking and sudden cardiac death. Adjusted estimates of the relative risk (RR) had to be available with the 95% confidence intervals (CIs) in the publication. No language restrictions were employed in the search or study selection. A list of the excluded studies can be found in Supplementary Table 3.

### Data extraction

The following data were extracted from each study: The first author’s last name, publication year, country where the study was conducted, study period, sample size, number of cases and participants, subgroup, relative risks and 95% confidence intervals for smokers versus nonsmokers and variables adjusted for in the analysis. DA conducted the data extraction and it was checked for accuracy by SS. Any disagreements between the authors were resolved by discussion.

### Statistical methods

We calculated summary RRs and 95% CIs of sudden cardiac death comparing current, former and ever smokers with never smokers using the random-effects model by DerSimonian and Laird [26] which takes into account both within and between study variation (heterogeneity). The average of the natural logarithm of the relative risks was estimated and the relative risk from each study was weighted using random effects weights. Studies that used non-current smokers (never + former smokers) as the reference category were analyzed separately to keep the reference category as clean as possible as there is evidence of increased risk also among former smokers. For studies that did not report results for ever smokers we pooled the RRs (95% CIs) for current and former smokers using a fixed-effects model to obtain risk estimates for ever smokers.

Heterogeneity between studies was evaluated using Q and I^2^ statistics [27]. I^2^ is a measure of how much of the heterogeneity that is due to between study variation rather than chance. I^2^-values of 25, 50 and 75% indicates low, moderate and high heterogeneity respectively. We conducted main analyses (all studies combined) and stratified by study characteristics such as sex, sample size, number of cases, geographic location, study quality and by adjustment for confounding factors (age, family history of coronary heart disease, alcohol, BMI, diabetes, hypertension, high cholesterol, prevalent coronary heart disease, use of QT prolonging drugs, use of digoxin, and physical activity) to investigate potential sources of heterogeneity.

Publication bias was assessed using Egger’s test [28] and Begg-Mazumdar’s test [29] and by inspection of funnel plots. The statistical analyses were conducted using the software package Stata, version 13.1 software (StataCorp, Texas, US).

## Results

**Fig. 1:**
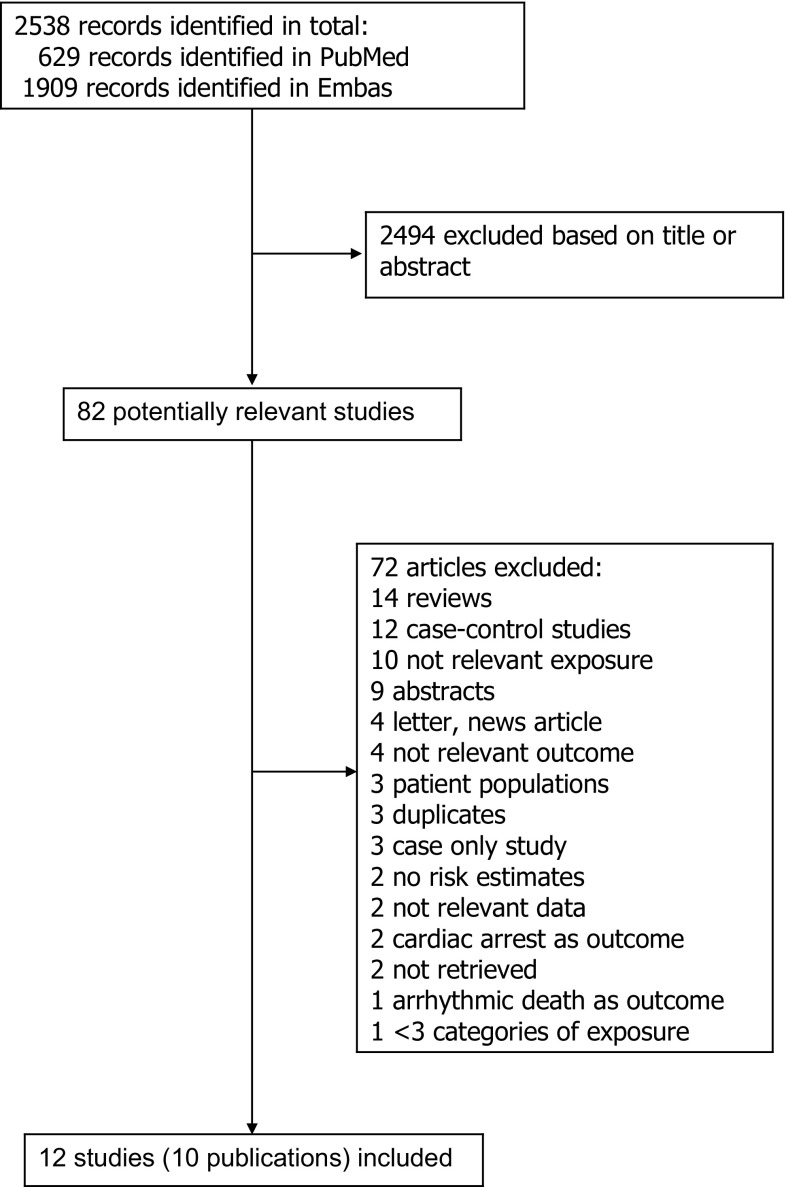
Flow-chart of study selection

**Fig. 2:**
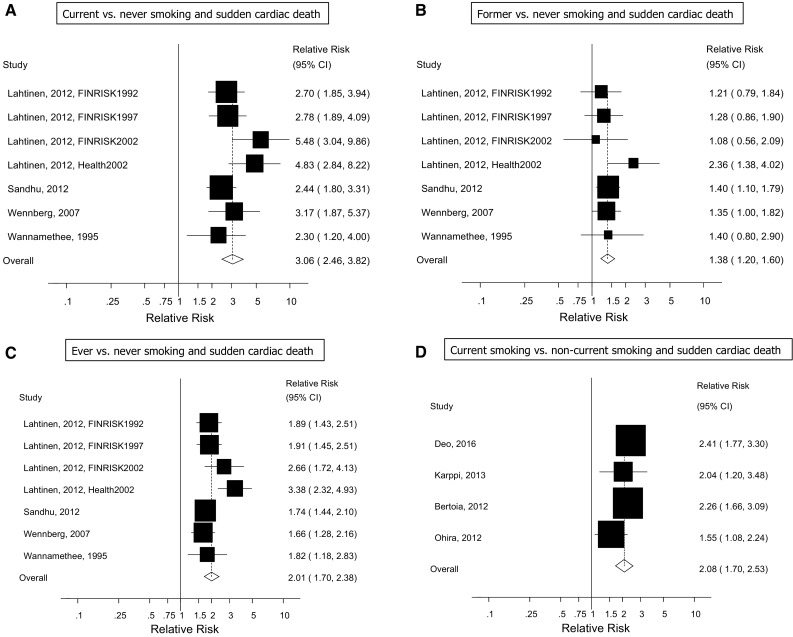
Smoking status and sudden cardiac death

We identified a total of 12 cohort studies (11 publications) [13–23] that were included in the meta-analysis (Fig. 1, Table 1). Nine of the studies were from Europe, three were from the USA, and one was from Japan. Seven studies (four publications) [14, 16–18] were included in the analysis of current, former, and ever smokers versus never smokers and risk of sudden cardiac death and included 1055 sudden cardiac deaths and 138273 participants. One publication included results from four different cohort studies [18]. The summary relative risk for current smokers versus never smokers was 3.06 (95% CIs 2.46–3.82, I^2^ = 40.5%, p_heterogeneity_ = 0.12, n = 7) (Fig. 2a) [14, 16–18], for former smokers it was 1.38 (95% CI 1.20–1.60, I^2^ = 0%, p_heterogeneity_ = 0.55, n = 7) (Fig. 2b) [14, 16–18], and for ever smokers it was 2.01 (95% CI 1.70–2.38, I^2^ = 54.1%, p_heterogeneity_ = 0.04, n = 7) (Fig. 2c) [14, 16–18]. There was no evidence of publication bias with Egger’s test or with Begg’s test (Supplementary Fig. 1) for current smokers (*p* = 0.12 and *p* = 0.14, respectively), former smokers (*p* = 0.85 and 0.24, respectively) or for ever smokers (*p* = 0.11 and 0.14, respectively). Among four studies [20–23] (1061 cases, 181,679 participants) that analyzed the association between current smoking and sudden cardiac death using non-current (never + former) smokers as the reference category the summary RR was 2.08 (95% CI: 1.70–2.53, I^2^ = 17.5%, p_heterogeneity_ = 0.30) (Fig. 2d).

**Table 1:** Prospective studies of smoking and sudden cardiac death

First author, publication year, country	Study name or description	Study period	Number of participants, sex, age, number of sudden cardiac deaths	Type of Smoking, subgroup	Comparison	Relative risk (95% confidence interval)	Adjustment for confounders
Wannamethee G et al., 1995, United Kingdom	British Regional Heart Study	1978–1980 —NA, 8 years follow-up	7735 men, age 40–59 years: 117 sudden cardiac deaths	Smoking status	Never Former Current	1.0 1.4 (0.8–2.9) 2.3 (1.2–4.0)	Age
Jouven X et al., 1999, France	Paris Prospective Study 1	1967–1972–1994, 23 years follow-up	7746 men, age 43-52 years: 118 sudden deaths	Smoking	Per 10.5 g/d	1.79 (1.31–2.40)	Age, BMI, DM, heart rate, SBP, cholesterol, TG, parental MI, parental sudden death
Wennberg P et al., 2007, Sweden	Vasterbotten Intervention Program and the Northern Sweden MONICA study	1986, 1990, 1994, 1999–1985–1999, ~ 4 years follow-up	Nested case–control study: 93 sudden cardiac deaths 1798 controls	Tobacco use	Never used tobacco	1.00	Age, sex, BMI, leisure-time physical activity, education + cholesterol
					Never smoker/former snuff	0.71 (0.36–1.43)	
					Never smoker/current snuff	0.90 (0.52–1.56)	
					Former smoker/never snuff	1.22 (0.85–1.74)	
					Former smoker/former snuff	1.39 (0.88–2.18)	
					Former smoker/current snuff	1.38 (0.89–2.14)	
					Current smoker/no current snuff	2.78 (2.05-3.78)	
					Current smoker/current snuff	2.33 (1.40–3.88)	
					Never used tobacco	1.00	
					Never smoker/former snuff	0.66 (0.32–1.34)	
					Never smoker/current snuff	0.82 (0.46–1.43)	
					Former smoker/never snuff	1.18 (0.82–1.70)	
					Former smoker/former snuff	1.34 (0.84–2.12)	
					Former smoker/current snuff	1.25 (0.80–1.96)	
					Current smoker/no current snuff	2.60 (1.91–3.54)	
					Current smoker/current snuff	2.14 (1.28–3.60)	
Laukkanen JA et al., 2010, Finland	Kuopio Ischemic Heart Disease Risk Factor Cohort	1984–1989 –2005, 17 years follow-up	2368 men, age 42–60 years: 146 sudden cardiac deaths	Cigarette smoking	Per 10 pack-years	1.27 (1.19–1.36)	Age, alcohol, hs-CRP, LDL cholesterol, HDL cholesterol, WHR, maximal heart rate during exercise
Sandhu RK et al., 2012, USA	Nurses’ Health Study	1980–2010, 30 years follow-up	101,018 women, age 34–59 years: 351 sudden cardiac deaths	Smoking status	Never	1.00	Age, DM, hypertension, hypercholesterolemia, BMI, alcohol, physical activity, menopausal status, postmenopausal hormone use, aspirin, multivitamin use, vitamin E, FH—MI
					Former	1.40 (1.10–1.79)	
					Current	2.44 (1.80–3.31)	
				Cigarettes per day	Current, 1–14 cig/d	1.84 (1.16–2.92)	
					Current, 15–24	2.62 (1.74–3.94)	
					Current, ≥ 25	3.30 (2.04–5.33)	
				Duration of smoking	Never	1.00	
					<12 years	1.35 (0.92–1.98)	
					13-< 24	1.02 (0.69–1.52)	
					25-< 35	1.48 (1.05–2.08)	
					≥35	2.22 (1.71–2.90)	
				Smoking cessation, no CHD at time of SCD	Current	1.00	
					<5 years	0.47 (0.24–0.92)	
					5-< 10	0.63 (0.35–1.14)	
					10-< 15	0.43 (0.21–0.87)	
					15-< 20	0.52 (0.28–0.98)	
					≥20	0.45 (0.30–0.68)	
					Never	0.41 (0.29–0.58)	
				Smoking cessation, CHD at time of SCD	Current	1.00	
					<5 years	2.14 (0.91–5.03)	
					5 < 10	1.25 (0.49–3.19)	
					10-< 15	1.61 (0.65–3.99)	
					15-< 20	0.53 (0.17–1.64)	
					≥20	0.59 (0.26–1.35)	
					Never	0.51 (0.24–1.10)	
Bertoia ML et al., 2012, USA	Women’s Health Initiative	1993–1998–2009, 10.8 years follow-up	161,808 women, age 50–79 years: 418 sudden cardiac deaths	Smoking status	Never, former Current	2.26 (1.66–3.09)	Age, race, total family income, resting pulse, BMI, WHR, CHD (excluding MI), MI, heart failure, atrial fibrillation, diabetes mellitus, carotid artery disease, hypertension
Ohira T et al., 2012, Japan	Circulatory Risk in Communities Study	1975–2005, ~ 3.5 years follow-up	26,870: nested case–control study: 239 sudden cardiac deaths 717 controls	Smoking	Never, former Current	1.55 (1.08–2.24)	Hypertension, diabetes, hyperlipidemia, excess ethanol intake, BMI, heart rate, atrial fibrillation, SVPC/VPC, major ST-T abnormalities, minor ST-T abnormalities, prolonged PQ duration, wide QRS, left high amplitude R wave, abnormal Q wave
Lahtinen AM et al., 2012, Finland	FINRISK 1992	1992–2008, ~ 16 years follow-up	5345 men and women, mean age 44.3 years: 129 sudden cardiac deaths	Smoking status	Never	1.00	Age, sex, geographic region, HDL/TC ratio, SBP, BMI, diabetes, physical activity, prevalent CHD, QT-prolonging drug, digoxin
					Former	1.21 (0.79–1.84)	
					Current	2.70 (1.85–3.94)	
Lahtinen AM et al., 2012, Finland	FINRISK 1997	1997–2008, ~ 11 years follow-up	7672 men and women, mean age 48.4 years: 178 sudden cardiac deaths	Smoking status	Never	1.00	Age, sex, geographic region, HDL/TC ratio, SBP, BMI, diabetes, physical activity, prevalent CHD, QT-prolonging drug, digoxin
					Former	1.28 (0.86–1.90)	
					Current	2.78 (1.89–4.09)	
Lahtinen AM et al., 2012, Finland	FINRISK 2002	2002–2008, ~ 6 years follow-up	8212 men and women, mean age 48.0 years: 75 sudden cardiac deaths	Smoking status	Never	1.00	Age, sex, geographic region, HDL/TC ratio, SBP, BMI, diabetes, physical activity, prevalent CHD, QT-prolonging drug, digoxin
					Former	1.08 (0.56–2.09)	
					Current	5.48 (3.04–9.86)	
Lahtinen AM et al., 2012, Finland	Health 2000	2000–2008, ~ 8 years follow-up	6400 men and women, mean age 53.0 years: 112 sudden cardiac deaths	Smoking status	Never	1.00	Age, sex, geographic region, HDL/TC ratio, SBP, BMI, diabetes, physical activity, prevalent CHD, QT-prolonging drug, digoxin
					Former	2.36 (1.38–4.02)	
					Current	4.83 (2.84–8.22)	
Karppi J et al., 2013, Finland	Kuopio Ischemic Heart Disease Risk Factor Cohort	1984–1989 –2008, 15.9 years follow-up	1031 men, age 46–65 years: 59 sudden cardiac deaths	Smoking	Never, former	1.00	Age, SBP, diabetes, waist circumference, alcohol, education, prevalent CHD, CHF, MI, hypertension, serum beta-carotene, LDL cholesterol, triglycerides, hs-CRP
					Current	2.04 (1.20–3.48)	
Deo R et al., 2016, USA	Atherosclerosis Risk in Communities Study (ARIC)	1989-1990 - 1992-1993 - NA, 14.2 years follow-up	13,677 men and women, mean age 54 years and 4207 men and women, mean age 72 years: 345 sudden cardiac deaths	Smoking status	Never/former	1.00	Age, sex, race, SBP, antihypertensive medication use, DM, potassium quadratic potassium term, albumin, HDL cholesterol, eGFR, QT interval
					Current	2.41 (1.77–3.30)	

*BMI* Body mass index, *CHD* coronary heart disease, *CHF* congestive heart failure, *DM* diabetes mellitus, *eGFR* estimated glomerular filtration rate, *FH* family history, *HDL* high-density lipoprotein, *hsCRP* high-sensitivity C-reactive protein, *LDL* low-density lipoprotein, *MI* myocardial infarction, *NA* not available, *SBP* systolic blood pressure, *SVPC* supraventricular premature complexes, *TC* total cholesterol, *TG* triglycerides, *VPC* ventricular premature complexes, *WHR* waist-to-hip ratio

**Fig. 3:**
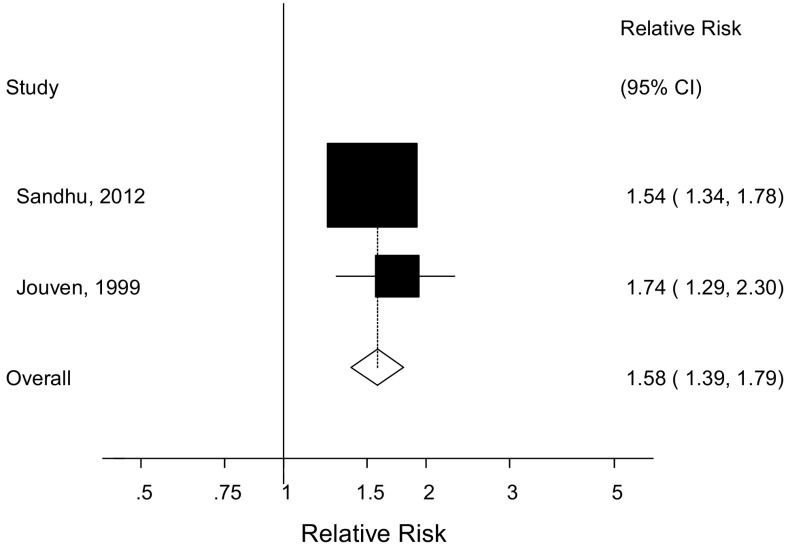
Cigarettes per day and sudden cardiac death, per 10 cigarettes per day

Only two studies [13, 17] were included in the dose–response analysis and included 469 sudden cardiac deaths and 108,764 participants. The summary RR per 10 cigarettes per day was 1.58 (1.39–1.79, I^2^ = 0%, p_heterogeneity_ = 0.46) (Fig. 3). Nonlinear analyses were not possible because only one of the studies [17] reported categorical data.

### Subgroup and sensitivity analyses and study quality

There were positive associations in all subgroup analyses, defined by duration of follow-up, sex, geographic location, number of cases, study quality and adjustment for confounding factors (including age, family history of coronary heart disease, alcohol, BMI, diabetes, hypertension, high cholesterol, prevalent coronary heart disease, use of QT prolonging drugs, use of digoxin, and physical activity) (Table 2). With meta-regression analyses there was little evidence that the results differed between these subgroups. Only in the subgroup analysis stratified by duration of follow-up among ever versus never smokers was there between-subgroup heterogeneity, p_heterogeneity_ = 0.04, with a stronger association among studies with a shorter compared to a longer duration of follow-up (2.57, 95% CI: 1.80–3.68 vs. 1.78, 95% CI 1.58–2.01, respectively).

**Table 2:** Subgroup analyses of smoking status and sudden cardiac death

	Current Smoking					Former smoking				
	*n*	RR (95% CI)	*I*^*2*^ (%)	*P*_h_^1^	*P*_h_^2^	*n*	RR (95% CI)	*I*^*2*^ (%)	*P*_h_^1^	*P*_h_^2^
All studies	7	3.06 (2.46–3.82)	40.5	0.12		7	1.38 (1.20–1.60)	0	0.55	
*Follow*-*up*										
< 10 years	3	3.97 (2.37–6.64)	58.6	0.09	0.10	3	1.58 (0.99–2.53)	43.8	0.17	0.37
≥ 10 years	4	2.67 (2.21–3.22)	0	0.85		4	1.34 (1.14–1.57)	0	0.94	
*Sex*										
Men	1	2.30 (1.20–4.00)			0.20	1	1.40 (0.80–2.90)			0.91
Women	1	2.44 (1.80–3.31)				1	1.40 (1.10–1.79)			
Men and women	5	3.44 (2.62–4.51)	40.2	0.15		5	1.38 (1.12–1.71)	18.9	0.30	
*Geographic location*										
Europe	6	3.26 (2.54–4.20)	37.2	0.16	0.35	6	1.38 (1.15–1.64)	0	0.42	0.91
America	1	2.44 (1.80–3.31)				1	1.40 (1.10–1.79)			
Asia	0					0				
*Number of cases*										
< 100	2	4.10 (2.40–7.01)	45.8	0.18	0.15	2	1.30 (0.99–1.71)	0	0.55	0.86
100-< 150	3	3.10 (2.05–4.67)	51.4	0.13		3	1.56 (1.03–2.38)	47.1	0.15	
≥150	2	2.57 (2.02–3.26)	0	0.60		2	1.37 (1.11–1.68)	0	0.71	
Study quality										
0–3 stars	0				0.18	0				0.90
4–6 stars	2	2.41 (1.84–3.16)	0	0.86		2	1.40 (1.11–1.76)	0	0.99	
7–9 stars	5	3.44 (2.62–4.51)	40.2	0.15		5	1.38 (1.12–1.71)	18.9	0.30	
*Adjustment for confounding factors*										
Age										
Yes	7	3.06 (2.46–3.82)	40.5	0.12	NC	7	1.38 (1.20–1.60)	0	0.55	NC
No	0					0				
*Family history of CHD*										
Yes	1	2.44 (1.80–3.31)			0.35	1	1.40 (1.10–1.79)			0.91
No	6	3.26 (2.54–4.20)	37.2	0.16		6	1.38 (1.15-1.64)	0	0.42	
*Alcohol*										
Yes	1	2.44 (1.80–3.31)			0.35	1	1.40 (1.10–1.79)			0.91
No	6	3.26 (2.54–4.20)	37.2	0.16		6	1.38 (1.15–1.64)	0	0.42	
*BMI*										
Yes	6	3.18 (2.49–4.05)	46.6	0.10	0.46	6	1.38 (1.19–1.60)	0	0.42	0.97
No	1	2.30 (1.20–4.00)				1	1.40 (0.80–2.90)			
*Diabetes*										
Yes	5	3.22 (2.42–4.28)	57.1	0.05	0.62	5	1.40 (1.14–1.70)	18.6	0.30	0.88
No	2	2.76 (1.85–4.10)	0	0.43		2	1.36 (1.04–1.78)	0	0.92	
*Hypertension*										
Yes	1	2.44 (1.80–3.31)			0.35	1	1.40 (1.10–1.79)			0.91
No	6	3.26 (2.54–4.20)	37.2	0.16		6	1.38 (1.15–1.64)	0	0.42	
*Blood cholesterol*										
Yes	5	3.22 (2.42–4.28)	57.1	0.05	0.63	5	1.40 (1.14–1.70)	18.6	0.30	0.88
No	2	2.76 (1.85–4.10)	0	0.43		2	1.36 (1.04–1.78)	0	0.92	
*Prevalent coronary heart disease*										
Yes	4	3.55 (2.52–5.00)	54.9	0.08	0.24	4	1.40 (1.03–1.91)1	38.9	0.18	0.98
No	3	2.55 (2.01–3.25)	0	0.66		3	1.38 (1.15–1.66)	0	0.98	
*QT prolonging drug use*										
Yes	4	3.55 (2.52–5.00)	54.9	0.08	0.24	4	1.40 (1.03–1.91)	38.9	0.18	0.98
No	3	2.55 (2.01–3.25)	0	0.66		3	1.38 (1.15–1.66)	0	0.98	
*Digoxin*										
Yes	4	3.55 (2.52–5.00)	54.9	0.08	0.24	4	1.40 (1.03–1.91)	38.9	0.18	0.98
No	3	2.55 (2.01–3.25)	0	0.66		3	1.38 (1.15–1.66)	0	0.98	
*Physical activity*										
Yes	6	3.18 (2.49–4.05)	46.6	0.10	0.46	6	1.38 (1.19–1.60)	0	0.42	0.97
No	1	2.30 (1.20-4.00)				1	1.40 (0.80–2.90)			

	Ever smoking				
	*n*	RR (95% CI)	*I*^*2*^ (%)	*P*_h_^1^	*P*_h_^2^
All studies	7	2.01 (1.70–2.38)	54.1	0.04	
*Follow*-*up*					
< 10 years	3	2.57 (1.80–3.68)	54.8	0.11	0.04
≥10 years	4	1.78 (1.58–2.01)	0	0.86	
*Sex*					
Men	1	1.82 (1.18–2.83)			0.47
Women	1	1.74 (1.44–2.10)			
Men and women	5	2.15 (1.69–2.72)	64.3	0.02	
*Geographic location*					
Europe	6	2.09 (1.70–2.57)	56.3	0.04	0.49
America	1	1.74 (1.44-2.10)			
Asia	0				
*Number of cases*					
< 100	2	2.03 (1.29–3.21)	69.5	0.07	0.68
100-< 150	3	2.26 (1.54–3.32)	70.7	0.03	
≥ 150	2	1.79 (1.53–2.09)	0	0.58	
*Study quality*					
0–3 stars	0				0.41
4–6 stars	2	1.75 (1.47–2.08)	0	0.85	
7–9 stars	5	2.15 (1.69–2.72)	64.3	0.02	
*Age*					
Yes	7	2.01 (1.70–2.38)	54.1	0.04	NC
No	0				
*Family history of CHD*					
Yes	1	1.74 (1.44–2.10)			0.49
No	6	2.09 (1.70–2.57)	56.3	0.04	
*Alcohol*					
Yes	1	1.74 (1.44–2.10)			0.49
No	6	2.09 (1.70–2.57)	56.3	0.04	
*BMI*					
Yes	6	2.04 (1.69–2.46)	61.6	0.02	0.74
No	1	1.82 (1.18–2.83)			
*Diabetes*					
Yes	5	2.15 (1.72–2.68)	65.0	0.02	0.35
No	2	1.70 (1.36–2.13)	0	0.72	
*Hypertension*					
Yes	1	1.74 (1.44–2.10)			0.49
No	6	2.09 (1.70–2.57)	56.3	0.04	
*Blood cholesterol*					
Yes	5	2.15 (1.72–2.68)	65.0	0.02	0.35
No	2	1.70 (1.36–2.13)	0	0.72	
*Prevalent coronary heart disease*					
Yes	4	2.32 (1.77–3.03)	61.8	0.05	0.14
No	3	1.72 (1.49–1.99)	0	0.93	
*QT prolonging drug use*					
Yes	4	2.32 (1.77–3.03)	61.8	0.05	0.14
No	3	1.72 (1.49–1.99)	0	0.93	
*Digoxin*					
Yes	4	2.32 (1.77–3.03)	61.8	0.05	0.14
No	3	1.72 (1.49–1.99)	0	0.93	
*Physical activity*					
Yes	6	2.04 (1.69–2.46)	61.6	0.02	0.74
No	1	1.82 (1.18–2.83)			

*n* denotes the number of studies

^1^P for heterogeneity within each subgroup

^2^P for heterogeneity between subgroups with meta-regression analysis

The association between smoking status and sudden cardiac death was robust in sensitivity analyses excluding one study at a time (Supplementary Fig. 1-3).

The mean (median) study quality scores were 8.0 (7.3) for the seven studies included in the analysis of smoking status and sudden cardiac death (Supplementary Table 4). There was no evidence of heterogeneity between subgroups when stratified by study quality scores.

## Discussion

To our knowledge, this is the first meta-analysis that summarize data on smoking status and risk of sudden cardiac death. There was a threefold increase in the risk of sudden cardiac death among current smokers, a 38% increase in the relative risk among former smokers and a twofold increase in the risk among ever smokers compared to never smokers. In a separate analysis of studies that compared current smokers with non-current (never + former) smokers there was a twofold increase in the risk. This is consistent with the main analysis as inclusion of former smokers together with never smokers would have contaminated the reference group and led to an underestimation of the true association between smoking and sudden cardiac death. There was a 58% increase in the relative risk per 10 cigarettes per day, however, only two studies were included in the dose–response analysis. The positive associations were observed across geographic location and sex, although the number of studies in some subgroups was modest.

The present systematic review and meta-analysis has some limitations that need to be discussed. Smokers often have less healthy lifestyles than persons never smokers, including more abdominal adiposity, less physically activity, and unhealthy diets. Some of the included studies adjusted for the most important confounding factors and the results persisted across all these subgroup analyses, and we found little evidence of heterogeneity between these subgroups. Only in the subgroup analysis stratified by duration of follow-up was there significant heterogeneity, and there was a weaker association among studies with a longer duration of follow-up compared to studies with a shorter duration of follow-up, although it’s unclear if this simply was a chance finding because of the many subgroup analyses that were conducted or if it could indicate some degree of regression dilution bias with misclassification of smoking exposure over time (for example if some of the smokers quit smoking during follow-up). Although the possibility that residual confounding partly could explain the results cannot be entirely excluded we consider this less likely as smoking appears to be one of the strongest risk factors for sudden cardiac deaths. Smoking was assessed by self-report, however, most studies have found a high correlation between self-reported smoking habits and biological markers of tobacco use [30] as well as strong associations with chronic disease outcomes [31]. Given the prospective study design any measurement errors or regression dilution bias would most likely have tended to lead to an underestimation of the association between smoking and sudden cardiac death. Although there was moderate heterogeneity in some of the analyses this appeared to the driven more by differences in the strength of the association than by differences in the direction of the association as all the studies found risk estimates in the direction of increased risk. Finally, few studies had investigated number of cigarettes and the duration of smoking cessation in relation to risk of sudden cardiac death, and any further studies should therefore aim to clarify the dose–response relationship between increasing number of cigarettes, duration of smoking or pack-years as well as the duration of smoking cessation and risk of sudden cardiac death.

The observed associations with a dose–response relationship between increasing number of cigarettes per day and increased risk of sudden cardiac death as well as a reduced risk of sudden cardiac deaths among former smokers compared to current smokers suggests an underlying biological relationship and several biological mechanisms could explain the observed associations. Approximately 80% of sudden cardiac deaths are ventricular tachyarrhythmias [2] and smokers have been found to be at increased risk of incident and recurrent ventricular tachyarrhythmias or ventricular fibrillation [32–34], which may be explained by altered ventricular recovery time dispersion indices [35]. Nicotine, which is one of the constituents of cigarettes, has been shown to induce a wide range of cardiac arrhythmias in animal studies, including transient sinus arrest, bradycardia, sinus tachychardia, atrial fibrillation, sinoatrial block, atrioventricular block and ventricular tachyarrhythmia [36]. Epidemiological studies have also found that smoking increases the risk of atrial fibrillation [37], which is a strong risk factor for sudden cardiac death [38]. Studies have also found that smoking increases blood pressure, resting heart rate and risk of type 2 diabetes [39–43], risk factors that have been associated with increased risk of sudden cardiac death [11, 13].

The present meta-analysis also has some strengths including the prospective design of the included studies (which avoids recall bias and reduces the potential for selection bias), the detailed subgroup and sensitivity analyses, the high study quality, and the increased sample size providing a more robust estimate of the association between smoking and risk of sudden cardiac death than any individual study. Our findings have important clinical and public health implications as cardiovascular diseases still is the leading cause of death worldwide, with coronary heart disease accounting for 8.9 million deaths globally in 2015 [1]. The current findings therefore provide further support for policies and interventions to prevent people from starting smoking in the first place and to aid smoking cessation among people who already smoke.

## Conclusion

This meta-analysis found a threefold increase in the relative risk of sudden cardiac death among current smokers, and a 38% increase in risk in former smokers. Any further studies should aim to analyze in more detail the association between number of cigarettes per day, duration of smoking, and pack-years as well as duration of smoking cessation and sudden cardiac death. Nevertheless, the current findings strongly support interventions and policies to curb the tobacco epidemic.

## Electronic supplementary material

Below is the link to the electronic supplementary material.

Supplementary material 1 (DOCX 87 kb)
