# Supplementary material for: Tobacco smoking and the risk of sudden cardiac death: a systematic review and meta-analysis of prospective studies
Source: Eur J Epidemiol. 2018 Feb 7;33(6):509–21. doi: 10.1007/s10654-017-0351-y (PMC5995997; doi:10.1007/s10654-017-0351-y)
Supplement: Supplementary file 1 — Supplementary material 1 (DOCX 87 kb) [file 10654_2017_351_MOESM1_ESM.docx]

Supplementary Table 1. Search terms in PubMed

| 1 | cigarette |
| --- | --- |
| 2 | cigarette[MeSH] |
| 3 | smoking |
| 4 | smoking[MeSH] |
| 5 | smoke |
| 6 | smoke[MeSH] |
| 7 | tobacco |
| 8 | tobacco[MeSH] |
| 9 | snus |
| 10 | snuff |
| 11 | snuff[MeSH] |
| 12 | "environmental tobacco smoke” |
| 13 | environmental tobacco smoke[MeSH] |
| 14 | “passive smoking” |
| 15 | passive smoking[MeSH] |
| 16 | “smoking cessation" |
| 17 | smoking cessation[MeSH] |
| 18 | "betel nut" |
| 19 | betel nut[MeSH] |
| 20 | bidi |
| 21 | pipe |
| 22 | cigar |
| 23 | cigar[MeSH] |
| 24 | "sudden cardiac death" |
| 25 | sudden cardiac death[MeSH] |
| 26 | "cardiac arrest" |
| 27 | cardiac arrest[MeSH] |
| 28 | 1 OR 2 OR 3 OR 4 OR 5 OR 6 OR 7 OR 8 OR 9 OR 10 OR 11 OR 12 OR 13 OR 14 OR 15 OR 16 OR 17 OR 18 OR 19 OR 20 OR 21 OR 22 OR 23 |
| 29 | 24 OR 25 OR 26 OR 27 |
| 30 | 28 AND 29 |

Supplementary Table 2. Search terms in Embase

| 1 | cigarette |
| --- | --- |
| 2 | cigarette/ |
| 3 | smoking |
| 4 | smoking/ |
| 5 | smoke |
| 6 | smoke/ |
| 7 | tobacco |
| 8 | tobacco/ |
| 9 | snus |
| 10 | snus/ |
| 11 | snuff |
| 12 | snuff/ |
| 13 | environmental tobacco smoke |
| 14 | environmental tobacco smoke/ |
| 15 | passive smoking |
| 16 | passive smoking/ |
| 17 | smoking cessation |
| 18 | smoking cessation/ |
| 19 | betel nut |
| 20 | betel nut/ |
| 21 | bidi |
| 22 | bidi/ |
| 23 | Pipe |
| 24 | pipe/ |
| 25 | cigar |
| 26 | cigar/ |
| 27 | sudden cardiac death |
| 28 | sudden cardiac death/ |
| 29 | cardiac arrest |
| 30 | cardiac arrest/ |
| 31 | 1 OR 2 OR 3 OR 4 OR 5 OR 6 OR 7 OR 8 OR 9 OR 10 OR 11 OR 12 OR 13 OR 14 OR 15 OR 16 OR 17 OR 18 OR 19 OR 20 OR 21 OR 22 OR 23OR 24 OR 25 OR 26 |
| 32 | 27 OR 28 OR 29 OR 30 |
| 33 | 31 AND 32 |

Supplementary Table 3. List of excluded studies and exclusion reason

| Exclusion reason | Reference number |
| --- | --- |
| <3 categories of cigarettes/day | (1) |
| Abstract only publication | (2-10) |
| Cardiac arrest as outcome | (11;12) |
| Case-control study | (13-24) |
| Case only study | (25-27) |
| Duplicates | (28-30) |
| Letter, news articles | (31-34) |
| No risk estimates | (35;36) |
| Not relevant data | (37;38) |
| Not relevant exposure | (39-48) |
| Not relevant outcome | (49-52) |
| Not retrieved | (53;54) |
| Patient populations | (55-57) |
| RCT - arrhythmic death as outcome | (58) |
| Review | (59-72) |

Reference List

1. Kagan A, Yano K, Reed DM, MacLean CJ. Predictors of sudden cardiac death among Hawaiian-Japanese men. Am J Epidemiol 1989;130:268-77.

2. Uehata A, Kurita A, Takase B et al. Heart rate, ischaemic heart disease, and sudden cardiac death in middle-aged British men. Journal of Cardiology 23 (SUPPL 37) (pp 89-93), 1993;23:89-93.

3. Sandhu RK, Jimenez M, Chiuve SE, Kenfield SA, Tedrow UB, Albert CM. Smoking, smoking cessation and risk of sudden cardiac death in women. Circulation Conference: American Heart Association's Scientific Sessions 2011;124:A15980.

4. Hagnas M, Kurl S, Rauramaa R, Makikallio TH, Laukkanen JA. Sudden cardiac death risk assessment, combining cardiorespiratory fitness with cardiovascular risk factors. European Heart Journal Conference: ESC Congress 2012;FP Nr. 3982.

5. Adabag S, Lopez F, Alonso A et al. Risk of sudden cardiac death in obese individuals: The atherosclerosis risk in communities (ARIC) study. Heart Rhythm Conference: 33rd Annual Scientific Sessions of the Heart Rhythm Society, Heart Rhythm 2012;9:S111-S112.

6. Hata J, Ninomiya T, Hirakawa Y et al. Trends in stroke, coronary heart disease, and cardiovascular risk factors during the past half century in a Japanese community: The Hisayama study, 1961-2009. Cerebrovascular Diseases Conference: 22nd European Stroke Conference London United Kingdom Conference Publication: 2013;35:140.

7. Adabag S, Lopez FL, Alonso A et al. Abdominal obesity is an independent risk factor for sudden cardiac death in non-smoking individuals: The atherosclerosis risk in communities study. Circulation Conference: American Heart Association 2013;128:A15872.

8. Pillon F. Sudden cardiac death risk factors identification in hypertensive patients. Fundamental and Clinical Pharmacology Conference: 17th Annual Meeting of French Society of Pharmacology and Therapeutics, the 80th Annual Meeting of Society of Physiology, the 34th Pharmacovigilance Meeting, the 14th APNET Seminar and the 11th CHU CIC Me 2013;27:113.

9. Gacon PH. Sudden cardiac death risk factor identification in hypertensive patients. European Heart Journal: Acute Cardiovascular Care Conference: Acute Cardiovascular Care 2014;October.

10. Olson K, Ahmad F, Bogle B, Ning H, Goldberger J, Lloyd-Jones D. Sudden cardiac death risk distribution in the united states population: Results from the national health and nutrition examination survey (nhanes), 2005-2012. Journal of the American College of Cardiology Conference: 66th Annual Scientific Session of the American College of Cardiology and i2 Summit: Innovation in Intervention, ACC 17 United States 2017;69:1739.

11. Thorgeirsson G, Thorgeirsson G, Sigvaldason H, Witteman J. Risk factors for out-of-hospital cardiac arrest: the Reykjavik Study. Eur Heart J 2005;26:1499-505.

12. Ohlsson MA, Kennedy LMA, Juhlin T, Melander O. Midlife risk factor exposure and incidence of cardiac arrest depending on cardiac or non-cardiac origin. Int J Cardiol 2017;240:398-402.

13. Yang J, Teehan D, Farioli A, Baur DM, Smith D, Kales SN. Sudden cardiac death among firefighters <=45 years of age in the United States. American Journal of Cardiology 1962;112:1962-7.

14. Siscovick DS, Weiss NS, Fox N. Moderate alcohol consumption and primary cardiac arrest. American Journal of Epidemiology 1986;123:499-503.

15. Cosin-Aguilar J, ndres-Conejos F, Hernandiz-Martinez A, Solaz-Minguez J, Marrugat J, Bayes-De-Luna A. Effect of smoking on sudden and premature death. J Cardiovasc Risk 1995;2:345-51.

16. Escobedo LG, Zack MM. Comparison of sudden and nonsudden coronary deaths in the United States. Circulation 1996;93:2033-6.

17. Escobedo LG, Caspersen CJ. Risk factors for sudden coronary death in the United States. Epidemiology 1997;8:175-80.

18. Sexton PT, Walsh J, Jamrozik K, Parsons R. Risk factors for sudden unexpected cardiac death in Tasmanian men. Aust N Z J Med 1997;27:45-50.

19. Owada M, Aizawa Y, Kurihara K, Tanabe N, Aizaki T, Izumi T. Risk factors and triggers of sudden death in the working generation: an autopsy proven case-control study. Tohoku J Exp Med 1999;189:245-58.

20. Kaikkonen KS, Kortelainen ML, Huikuri HV. Comparison of risk profiles between survivors and victims of sudden cardiac death from an acute coronary event. Ann Med 2009;41:120-7.

21. Tyynela P, Goebeler S, Ilveskoski E et al. Birthplace predicts risk for prehospital sudden cardiac death in middle-aged men who migrated to metropolitan area: The Helsinki Sudden Death Study. Ann Med 2009;41:57-65.

22. van Teeffelen WM, de Beus MF, Mosterd A et al. Risk factors for exercise-related acute cardiac events. A case-control study. Br J Sports Med 2009;43:722-5.

23. Chow KM, Szeto CC, Kwan BC, Chung KY, Leung CB, Li PK. Factors associated with sudden death in peritoneal dialysis patients. Perit Dial Int 2009;29:58-63.

24. Hsue PY, McManus D, Selby V et al. Cardiac arrest in patients who smoke crack cocaine. Am J Cardiol 2007;99:822-4.

25. Silva AC, Santos L, nis-Oliveira RJ, Magalhaes T, Santos A. Sudden Cardiac Death in Young Adult. Cardiovascular Toxicology 14 (4) (pp 379-386), 2014;2014.

26. Burke AP, Farb A, Pestaner J et al. Traditional risk factors and the incidence of sudden coronary death with and without coronary thrombosis in blacks. Circulation 2002;105:419-24.

27. Maruyama M, Ohira T, Imano H et al. Trends in sudden cardiac death and its risk factors in Japan from 1981 to 2005: the Circulatory Risk in Communities Study (CIRCS). BMJ Open 2012;2:e000573.

28. Jouven X, Zureik M, Desnos M, Guerot C, Ducimetiere P. Resting heart rate as a predictive risk factor for sudden death in middle-aged men. Cardiovasc Res 2001;50:373-8.

29. Albert CM, Chae CU, Grodstein F et al. Prospective study of sudden cardiac death among women in the United States. Circulation 2003;107:2096-101.

30. Chiuve SE, Fung TT, Rexrode KM et al. Adherence to a low-risk, healthy lifestyle and risk of sudden cardiac death among women. JAMA 2011;306:62-9.

31. Golden J, Kitahata M, Lieu T. Smoking as a risk factor for recurrence of sudden cardiac arrest. New England Journal of Medicine 314 (25) (pp 1641-1642), 1986;1986.

32. Golden J, Kitahata M, Lieu T, Wilson C, Steinmann W. Smoking as a risk factor for recurrence of sudden cardiac arrest. N Engl J Med 1986;314:1641-2.

33. Risk factors linked to sudden cardiac death in women. Heart Advis 2003;6:2.

34. Smoking raises the risk of sudden death in women. Harv Heart Lett 2013;23:8.

35. Wannamethee G, Shaper AG. Alcohol and sudden cardiac death. British Heart Journal 68 (5) (pp 443-448), 1992;68:443-8.

36. Srivatsa UN, Swaminathan K, Sithy Athiya MK, Amsterdam E, Shantaraman K. Sudden cardiac death in South India: Incidence, risk factors and pathology. Indian Pacing and Electrophysiology Journal 2016;16:121-5.

37. Cullen P, Schulte H, Assmann G. Smoking, lipoproteins and coronary heart disease risk. Data from the Munster Heart Study (PROCAM). Eur Heart J 1998;19:1632-41.

38. Turner MC, Cohen A, Burnett RT et al. Interactions between cigarette smoking and ambient PM<inf>2.5</inf> for cardiovascular mortality. Environmental Research 2017;154:304-10.

39. Shaper AG, Wannamethee G, Macfarlane PW, Walker M. Heart rate, ischaemic heart disease, and sudden cardiac death in middle-aged British men. British Heart Journal 1993;70:49-55.

40. Okin PM, Kjeldsen SE, Julius S, Dahlof B, Devereux RB. Racial differences in sudden cardiac death among hypertensive patients during antihypertensive therapy: the LIFE study. Heart rhythm 2012;9:531-7.

41. Laukkanen JA, Jennings JR, Kauhanen J, Makikallio TH, Ronkainen K, Kurl S. Relation of systemic blood pressure to sudden cardiac death. American Journal of Cardiology 2012;110:378-82.

42. Hurt RD, Weston SA, Ebbert JO et al. Myocardial infarction and sudden cardiac death in Olmsted County, Minnesota, before and after smoke-free workplace laws. Archives of Internal Medicine 2012;172:1635-41.

43. Havmoeller R, Reinier K, Teodorescu C et al. Elevated plasma free fatty acids are associated with sudden death: A prospective community-based evaluation at the time of cardiac arrest. Heart Rhythm 11 (4) (pp 691-696), 2014;11:691-6.

44. Bertoia ML, Triche EW, Michaud DS et al. Mediterranean and Dietary Approaches to Stop Hypertension dietary patterns and risk of sudden cardiac death in postmenopausal women1-3. American Journal of Clinical Nutrition 2014;99:344-51.

45. Reinier K, Marijon E, Uy-Evanado A et al. The association between atrial fibrillation and sudden cardiac death: The relevance of heart failure. JACC: Heart Failure 2014;2:221-7.

46. Vasiliadis I, Kolovou G, Mavrogeni S, Nair DR, Mikhailidis DP. Sudden cardiac death and diabetes mellitus. Journal of Diabetes and its Complications 2014;28:573-9.

47. Adabag S, Huxley RR, Lopez FL et al. Obesity related risk of sudden cardiac death in the atherosclerosis risk in communities study. Heart 2015;3-215.

48. Park SK, Tucker KL, O'Neill MS et al. Fruit, vegetable, and fish consumption and heart rate variability: The Veterans administration normative aging study. American Journal of Clinical Nutrition 2009;89:778-86.

49. Tonascia J, Szklo M, Goldberg R, Kennedy H. Predictors of ventricular fibrillation or cardiac arrest in patients hospitalized for acute myocardial infarction. Clinical Cardiology 1981;4:168-71.

50. Maas R, Schulze F, Baumert J et al. Asymmetric dimethylarginine, smoking, and risk of coronary heart disease in apparently healthy men: Prospective analysis from the population-based Monitoring of Trends and determinants in Cardiovascular Disease/Kooperative Gesundheitsforschung in der Region Augsburg study and experimental data. Clinical Chemistry 2007;53:693-701.

51. Subirana MT, Juan-Babot JO, Puig T et al. Specific characteristics of sudden death in a mediterranean Spanish population. American Journal of Cardiology 2011;107:622-7.

52. Puddu PE, Terradura VO, Mancini M, Zanchetti A, Menotti A. Typical and atypical coronary heart disease deaths and their different relationships with risk factors. The Gubbio residential cohort study. Int J Cardiol 2014;173:300-4.

53. Cupples LA, Gagnon DR, Kannel WB. Long- and short-term risk of sudden coronary death. Circulation 1992;85:I11-I18.

54. Friedman GD, Klatsky AL, Siegelaub AB. Predictors of sudden cardiac death. Circulation 1975;52:III164-III169.

55. Hallstrom AP, Cobb LA, Ray R. Smoking as a risk factor for recurrence of sudden cardiac arrest. N Engl J Med 1986;314:271-5.

56. Goldenberg I, Jonas M, Tenenbaum A et al. Current smoking, smoking cessation, and the risk of sudden cardiac death in patients with coronary artery disease. Arch Intern Med 2003;163:2301-5.

57. Sanchez JM, Greenberg SL, Chen J et al. Smokers are at markedly increased risk of appropriate defibrillator shocks in a primary prevention population. Heart Rhythm 2006;3:443-9.

58. Peters RW, Brooks MM, Todd L, Liebson PR, Wilhelmsen L. Smoking cessation and arrhythmic death: the CAST experience. The Cardiac Arrhythmia Suppression Trial (CAST) Investigators. J Am Coll Cardiol 1995;26:1287-92.

59. Kupari M, Koskinen P. Alcohol, cardiac arrhythmias and sudden death. Novartis Foundation symposium 1998;216:68-79.

60. Hershberg PI, Alexander S. Prevention and treatment of sudden cardiac death. Medical Clinics of North America 1972;56:625-31.

61. Paul O. Epidemiological and clinical aspects of sudden cardiac death. Triangle; the Sandoz journal of medical science 1973;12:17-20.

62. Meinertz T, Hofmann T, Zehender M. Can we predict sudden cardiac death? Drugs 1991;41:9-15.

63. Futterman LG, Lemberg L. Sudden cardiac death--preventable--reversible. American journal of critical care : an official publication, American Association of Critical-Care Nurses 1997;6:472-82.

64. Asplund K. Smokeless tobacco and cardiovascular disease. Prog Cardiovasc Dis 2003;45:383-94.

65. Rempher KJ. Cardiovascular sequelae of tobacco smoking. Crit Care Nurs Clin North Am 2006;18:13-20, xi.

66. Mehra R. Global public health problem of sudden cardiac death. J Electrocardiol 2007;40:S118-S122.

67. Bullen C. Impact of tobacco smoking and smoking cessation on cardiovascular risk and disease. Expert Rev Cardiovasc Ther 2008;6:883-95.

68. Gastaldelli A, Folli F, Maffei S. Impact of tobacco smoking on lipid metabolism, body weight and cardiometabolic risk. Curr Pharm Des 2010;16:2526-30.

69. Pipe AL, Eisenberg MJ, Gupta A, Reid RD, Suskin NG, Stone JA. Smoking cessation and the cardiovascular specialist: Canadian Cardiovascular Society position paper. Can J Cardiol 2011;27:132-7.

70. Smith JD, Clinard V. Diabetes and sudden cardiac death. U S Pharmacist 2013;38:38-42.

71. Sawyer KN, Lundbye JB. History of smoking: A form of ischemic preconditioning? Implications for surviving cardiac arrest. Resuscitation 2014;85:13-4.

72. Al-Khatib SM, Yancy CW, Solis P et al. 2016 AHA/ACC clinical performance and quality measures for prevention of sudden cardiac death: A report of the American College of Cardiology/American Heart Association Task Force on performance measures. Circulation: Cardiovascular Quality and Outcomes 2017;10:e000022.

Supplementary Table 4. Study quality of studies on smoking and sudden cardiac death

| Author, publication year | Represen-tativeness | Selection of non-exposed cohort | Exposure-ascertainment | Demonstration of outcome not present at start^1^ | Adjustment for one risk factor | Adjustment for any other factor | Assess-ment of outcome | Long enough follow-up^2^ | Adequacy of follow-up^3^ | Total score |
| --- | --- | --- | --- | --- | --- | --- | --- | --- | --- | --- |
| Wannamethee, 1995 | 1 | 1 | 0 | 0 | 1 | 0 | 1 | 1 | 1 | 6 |
| Wennberg, 2007 | 1 | 1 | 0 | 1 | 1 | 1 | 1 | 1 | 0 | 7 |
| Sandhu, 2012 | 0 | 1 | 0 | 1 | 1 | 1 | 1 | 1 | 0 | 6 |
| Lahtinen, 2012, FINRISK 1992 | 1 | 1 | 1 | 0 | 1 | 1 | 1 | 1 | 1 | 8 |
| Lahtinen, 2012, FINRISK 1997 | 1 | 1 | 1 | 0 | 1 | 1 | 1 | 1 | 1 | 8 |
| Lahtinen, 2012, FINRISK 2002 | 1 | 1 | 1 | 0 | 1 | 1 | 1 | 1 | 1 | 8 |
| Lahtinen, 2012, Health 2002 | 1 | 1 | 1 | 0 | 1 | 1 | 1 | 1 | 1 | 8 |

^1^ 1 point for exclusion of prevalent cardiovascular disease cases at baseline

^2^ 1 point for follow-up of ≥3 years

^3^ 1 point for loss-to-follow-up less than 10%

Supplementary Figure 1. Current versus never smokers and sudden cardiac death, sensitivity analysis excluding one study at a time

------------------------------------------------------------------------------

Study omitted | Estimate [95% Conf. Interval]

-------------------+----------------------------------------------------------

Lahtinen, 2012, FINRISK1992| 3.179374 2.4314852 4.1573029

Lahtinen, 2012, FINRISK1997| 3.1603429 2.4145293 4.136528

Lahtinen, 2012, FINRISK2002| 2.8188086 2.3525772 3.3774371

Lahtinen, 2012, Health2002| 2.8483689 2.3291273 3.4833667

Sandhu, 2012 | 3.2637665 2.5392151 4.195065

Wennberg, 2007 | 3.0747299 2.3821254 3.9687092

Wannamethee, 1995 | 3.177624 2.4942236 4.0482712

-------------------+----------------------------------------------------------

Combined | 3.0645271 2.4570967 3.8221233

------------------------------------------------------------------------------

Supplementary Figure 2. Former versus never smokers and sudden cardiac death, sensitivity analysis excluding one study at a time

------------------------------------------------------------------------------

Study omitted | e^coef. [95% Conf. Interval]

-------------------+----------------------------------------------------------

Lahtinen, 2012, FINRISK1992| 1.4083216 1.2082859 1.6414738

Lahtinen, 2012, FINRISK1997| 1.4002298 1.1996579 1.6343355

Lahtinen, 2012, FINRISK2002| 1.4010644 1.2087996 1.6239097

Lahtinen, 2012, Health2002| 1.3270988 1.1427467 1.5411915

Sandhu, 2012 | 1.3750424 1.150085 1.6440017

Wennberg, 2007 | 1.3940444 1.1828451 1.6429536

Wannamethee, 1995 | 1.382875 1.1929035 1.6030996

-------------------+----------------------------------------------------------

Combined | 1.3837267 1.1981157 1.5980924

------------------------------------------------------------------------------

Supplementary Figure 3. Ever versus never smokers and sudden cardiac death, sensitivity analysis excluding one study at a time

------------------------------------------------------------------------------

Study omitted | e^coef. [95% Conf. Interval]

-------------------+----------------------------------------------------------

Lahtinen, 2012, FINRISK1992| 2.0449266 1.6694506 2.5048511

Lahtinen, 2012, FINRISK1997| 2.0419333 1.6651816 2.5039258

Lahtinen, 2012, FINRISK2002| 1.9445319 1.6365155 2.3105216

Lahtinen, 2012, Health2002| 1.8312898 1.6365051 2.0492587

Sandhu, 2012 | 2.092263 1.7045116 2.568222

Wennberg, 2007 | 2.0919998 1.7234235 2.5394011

Wannamethee, 1995 | 2.038331 1.6872904 2.4624057

-------------------+----------------------------------------------------------

Combined | 2.0069682 1.6956168 2.3754904

------------------------------------------------------------------------------
